# Protective Effects of Mango Ginger (*Curcuma amada* Roxb.) Ethanol Extract against Carbon Tetrachloride–Induced Toxicity: In Vivo and In Silico Evidence

**DOI:** 10.1016/j.cdnut.2026.109506

**Published:** 2026-07-31

**Authors:** Sagor Saha, Abu Amer Hisham, Md. Mahmodul Islam, Mohammad Salim Hossain, Md. Monirul Islam

**Affiliations:** Department of Pharmacy, Faculty of Biological Sciences, Noakhali Science and Technology University, Noakhali, Bangladesh

**Keywords:** nutritional bioactive, inflammatory mediators, medicinal plants, functional foods, oxidative stress

## Abstract

**Background:**

Mango ginger [*Curcuma amada* Roxb. (CA)] is a rhizomatous plant used in preparing pickles and culinary dishes. It also has medicinal values, including anti-inflammatory, antioxidant, and antimicrobial activities.

**Objectives:**

The present study aims to investigate the protective effects of CA ethanol extract against carbon tetrachloride (CCl_4_)–induced damage.

**Methods:**

The CA ethanol extract was analyzed by gas chromatography–mass spectrometry (GC-MS). Mice were divided into 5 groups: control, CCl_4_ only [1 mL/kg body weight (BW)], CCl_4_ with CA (200 and 400 mg/kg BW), and CCl_4_ with silymarin (50 mg/kg BW). The hepatoprotective potential of CA in mice was evaluated by measuring the serum alanine transaminase (ALT), aspartate aminotransferase (AST), alkaline phosphatase (ALP), and bilirubin levels. Moreover, the lipid profile and kidney biomarkers were also evaluated. Histopathological changes in the liver and kidney were assessed using hematoxylin and eosin staining. The molecular docking of the CA-derived compounds and the absorption, distribution, metabolism, excretion, and toxicity (ADMET) profile of the selected compounds were also evaluated.

**Results:**

GC-MS identified 62 probable compounds in the extract. CA significantly (*P* < 0.05) reduced elevated liver biomarkers (ALT, AST, ALP, and bilirubin), kidney biomarkers (urea and creatinine), and lipid levels. These findings aligned with histopathological observations, in which CA reversed the toxic effects of CCl_4_ on liver and kidney cells. Molecular docking showed potent binding affinities for inflammatory, hepatoprotective, and oxidative targets. The bioactive compounds showed moderate ADMET properties.

**Conclusions:**

Our findings show that CA has potential protective effects that can be further studied to develop novel hepatoprotective and nephroprotective agents.

## Graphical abstract

**Figure:**
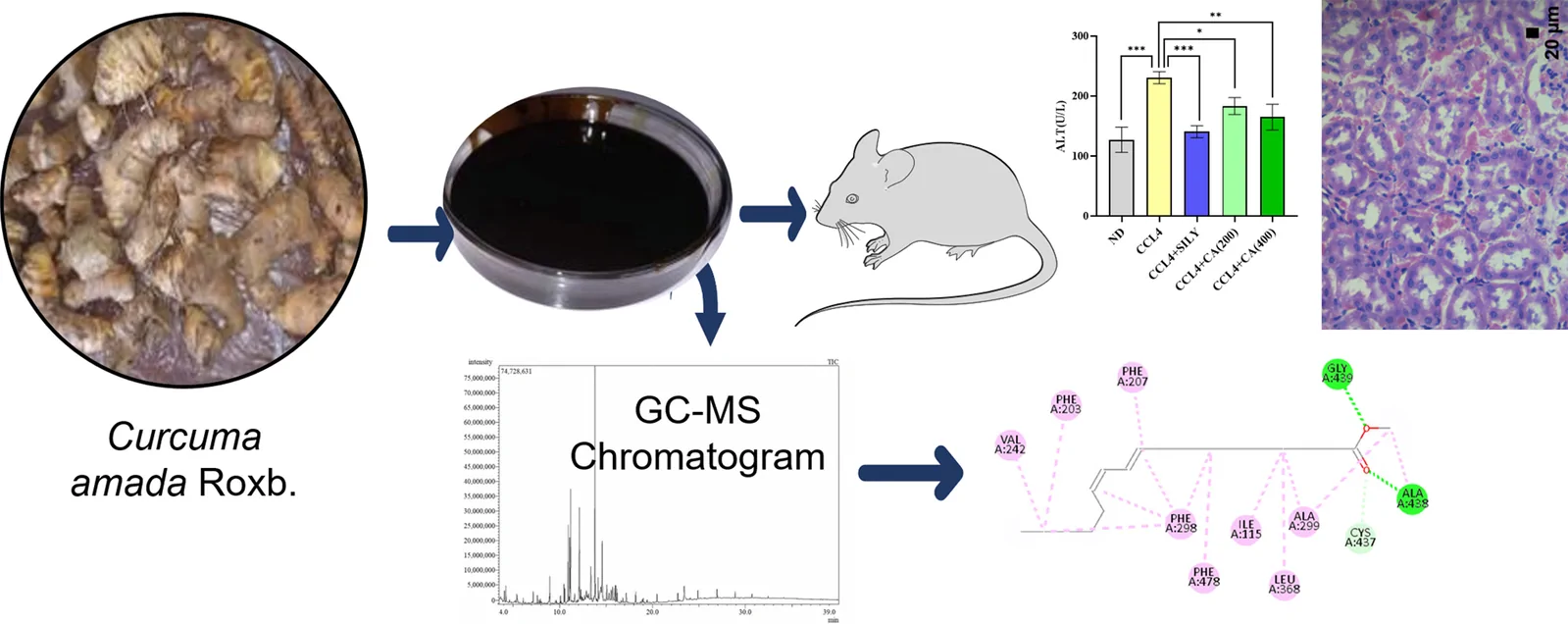

## Introduction

The liver is a fundamental organ in humans that performs essential functions, including metabolism, secretion, and storage. It can produce beneficial substances and neutralize detrimental chemicals [1]. The global problem of liver-related diseases remains a significant challenge to public health. The primary causes include obesity, metabolic disorders, medications, excessive alcohol consumption, infections, and autoimmune disorders [2,3]. Most hepatotoxic agents predominantly induce lipid peroxidation and other forms of oxidative injury to hepatic cells [4]. The kidney is a crucial organ primarily responsible for eliminating waste products. This organ performs multiple physiological functions, including the maintenance of homeostasis in body fluids, involvement in glucose metabolism, erythropoiesis, regulation of blood pressure, and the production of hormones and enzymes [5,6]. The excretion of xenobiotics primarily occurs via the kidneys, which are subjected to free radicals that elevate oxidative stress, leading to renal impairment. The WHO reports that liver illness constitutes ∼4% of worldwide mortality, resulting in 2 million deaths yearly, whereas ∼10% of the global population suffers from renal disease, leading to 1.3 million fatalities each year [4]. Carbon tetrachloride (CCl_4_) is a well-established experimental toxicant widely used to induce both hepatic and renal injury [7]. After metabolic activation, CCl_4_ generates highly reactive free radicals that initiate lipid peroxidation, inflammation, and cellular necrosis in multiple organs, particularly the liver and kidneys [8].

Medicinal plants have demonstrated benefit in treating several diseases; nevertheless, comprehensive safety and efficacy data remain insufficient. Silymarin is a traditional remedy for liver disease, purported to be safe and effective [9,10]. It has been reported to protect hepatic and renal tissues against CCl_4_-induced oxidative damage by inhibiting lipid peroxidation, enhancing endogenous antioxidant defense systems, and promoting cellular regeneration. Therefore, it is commonly employed as a reference standard for evaluating the protective efficacy of novel plant-derived therapeutic agents [11,12]. Current pharmacological interventions can mitigate many hepatic and renal disorders based on their primary etiologies and postpone the onset of end-stage liver and kidney failure. Nevertheless, pharmacological treatment has been unable to fully restore liver and kidney function. Many pharmaceuticals induce significant liver and kidney damage and are regarded as risk factors for these organs [13]. In this setting, alternative therapies are required to prevent or address hepatic and renal diseases [14]. Medicinal plants have historically been used in various countries to treat and prevent specific ailments [15,16].

*Curcuma amada* Roxb. (CA) is a distinct spice that resembles ginger (*Zingiber officinale*) in appearance but has a raw mango (*Mangifera indica*) flavor. It is a perennial rhizomatous aromatic herb from the Zingiberaceae family. Mango ginger rhizomes are fleshy, buff-colored, and divided into nodes and internodes. Scaly leaves at rhizome nodes are grouped in a circular manner, resembling growth rings with scars on the surface. The rhizomes have a raw-mango flavor and are peppery. As a result, it is a key component in pickles, preserves, sweets, sauces, salads, and curries [17]. Mango ginger is commonly used in Ayurvedic and Unani medicine as a digestive aid, diuretic, laxative, antiasthmatic, and expectorant [18]. The rhizome has antimicrobial, lipid-lowering, glucose-lowering, anthelmintic, antitubercular, and antipyretic activity [19].

According to prior research findings, CA exhibited strong antioxidant, anti-inflammatory, and antihyperlipidemic properties [18]. However, the hepatoprotective activity of this plant has not been widely studied. The objective of this work was to investigate the hepatoprotective and nephroprotective effects of CA against CCl_4_-induced toxicity in mouse models.

## Methods

### Preparation of plant extract

The rhizome was obtained from Chattogram, Bangladesh, on October 13, 2023. The plant was identified by an expert of the Bangladesh National Herbarium, and a specimen was stored for future reference (Accession number: DACB 137468). The plant was rinsed and purified using tap water to eliminate any particles or impurities. The plant was fragmented into small segments and left to air-dry for 25 d. The dry plant components (165 g) were ground into a powder, and the weight of the dried powder was measured (142 g). The resulting 142 g of dried CA powder was weighed and placed in an amber jar. It was soaked in 75% ethanol (320 mL of ethanol + 107 mL of deionized water) for 42 d. It was filtered using a Whatman filter paper, and the filtrate was collected. Later, the solvent was evaporated at 45 to 48°C (optimum temperature 37°C) and 90 to 95 rpm (optimum 55 rpm) by a rotatory vacuum evaporator for 8 h and collected. Then, 23.1503 g of extract was weighed using an electronic balance and covered with foil paper. After that, it was stored in the refrigerator.

### GC-MS analysis

The GC-MS analysis was performed on a SHIMADZU GC-MS TQ8040 (manufacturer: Shimadzu Corporation) equipped with an autosampler (AOC-20s) and autoinjector (AOC-20i) using a SH-Rxi-5Sil MS column (30 m × 0.25 mm; 0.25 μm). The helium was used as carrier gas at 1.72 mL/min flow rate; oven temperature was programmed from 80°C (hold time 2.00 min, raised at 5°C/min) to 150°C (hold time 5.00 min) and final temperature of 280°C (hold time 5.00 min). The bioactive compounds were identified based on retention time, MS fragment ions generated, and the percentage of these bioactive compounds was evaluated from the total peak area. The phytochemicals were identified by comparing their mass spectra with those of NIST08s, NIST08, and NIST14 libraries [20].

### Experimental animal

For this experiment, 35 mature male Swiss albino mice weighing 22 to 25 g were used. The mice used in this study were sourced from the Animal Resources Facility of the International Centre for Diarrheal Disease Research, Bangladesh (icddr,b). The mice were individually housed in metal enclosures with 12-h cycles of light and darkness, maintaining constant environmental and food conditions. The distinctive opening provided unhindered entry to uncontaminated and untainted potable water. The mice were fed an icddr,b-designed meal pellet that was composed of 53.85% carbohydrate, 19.7% protein, 15.75% fat, 4.2% fiber, and 6.5% ash [21]. After a 7-d period of acclimatization, the experiments were carried out. The mouse experiments were carried out in Bangladesh at the Department of Pharmacy, Noakhali Science and Technology University.

### Ethical clearance

The experimental techniques outlined in the current work underwent a thorough review and were approved by the Noakhali Science and Technology University Ethical Committee (NSTU/SCI/EC/2023/219). The experimental subjects were handled in accordance with the committee’s ethical guidelines.

### Experimental design

The animals were randomly divided into 5 experimental treatment groups of 7 animals each. Animals in group I served as the normal control, given water and a normal diet only. Groups II, III, IV, and V received an oral dose of CCl_4_ (1 mL/kg, by mouth every third day) for 4 wk, which has been shown to cause subchronic hepatotoxicity and nephrotoxicity in mice [22,23]. During the same experimental period, mice in groups III, IV, and V simultaneously received daily oral administration of silymarin [50 mg/kg body weight (BW, by mouth)], CA (200 mg/kg BW, by mouth), and CA (400 mg/kg BW, by mouth), respectively. Thus, the study employed a cotreatment design to evaluate the protective effects of the interventions against ongoing CCl_4_-induced toxicity. Silymarin-treated mice served as a positive control group because of silymarin’s proven role as a hepatoprotective agent [12]. The experimental treatments and group designs were as follows:

Group I (normal diet): for 4 wk, mice were given only water and a normal diet.

Group II (disease control): mice received CCl_4_ (1 mL/kg BW, 1:1 vol:vol olive oil mixture, by mouth) every third day for 4 wk.

Group III (standard group): for 4 wk, mice received CCl_4_ (1 mL/kg BW, by mouth every third day) + silymarin (50 mg/kg BW/d, by mouth).

Group IV (CA 200): for 4 wk, mice received CCl_4_ (1 mL/kg BW, by mouth every third day) + a daily dose of 200 mg/kg BW of CA.

Group V (CA 400): For 4 wk, mice received CCl4 (1 mL/kg BW, by mouth every third day) + a daily dose of 400 mg/kg BW of CA.

Animals were randomly allocated to the experimental groups before treatment initiation. However, allocation concealment was not implemented, and the principal investigator remained aware of the treatment assignments throughout the experiment. Consequently, complete investigator blinding was not achieved.

### Organ and serum collection

On the 29th day, the mice were weighed and briefly anesthetized using chloroform. By puncturing the hearts of each experimental animal, blood was collected and transferred into Eppendorf tubes. The tubes were then left at room temperature for 30 min to allow coagulation. Subsequently, the sample was centrifuged at 3000 rpm for 10 min at 4°C to obtain the blood serum. Subsequently, an additional Eppendorf tube was used to segregate the serum, which was then preserved at a temperature of −80°C until the biochemical analysis was conducted [24]. In the meantime, each mouse’s liver and kidney were surgically removed and placed in the saline solution (0.9% NaCl) right afterward. Finally, each mouse’s liver and kidney were weighed and preserved in 10% neutral buffered formalin prepared from commercial 37% formaldehyde solution for histological analysis [25]. The colorimetric measurement of alanine aminotransferase (ALT), serum aspartate transaminase (AST), alkaline phosphatase (ALP), total triglycerides (TGs), total cholesterol (TC), bilirubin, urea, and creatinine were measured using a semiautomatic Thermo Scientific Multiskan FC Microplate Photometer by Thermo Fisher Scientific with a commercially available colorimetric reagent.

### Statistical analysis

Statistical analyses were performed using GraphPad Prism software (version 8.00). The biochemical data were provided as mean ± SEM. The normality of the data distribution was evaluated using the Shapiro–Wilk test, and all datasets showed normal distribution (*P* > 0.05). Homogeneity of variances was evaluated using Levene’s test, which demonstrated equal variances among the groups for all analyzed parameters. Therefore, 1-way analysis of variance followed by Tukey’s multiple comparison post hoc test was applied for intergroup comparisons. Exact *P* values were calculated, and a *P* value of < 0.05 was considered statistically significant. A priori sample size calculation was not performed before the initiation of this study. The number of animals (*n* = 7 per group) was selected based on sample sizes commonly reported in comparable CCl_4_-induced hepatorenal toxicity studies and institutional experience with similar experimental models. Although this sample size was sufficient to detect statistically significant differences for several outcome measures, the absence of a formal power analysis is acknowledged as a methodological limitation.

### Histopathological examination

After purification, the water content in liver and kidney tissue samples is eliminated using an alcohol dehydration method. Thereafter, xylene was used for washing to remove alcohol and achieve tissue translucency. Thereafter, paraffin infiltration was performed at room temperature to solidify the tissue, enabling sectioning with a microtome. Paraffin blocks were sectioned to a thickness of 5 to 6 μm, and the slides were deparaffinized with xylene, followed by hematoxylin and eosin staining (at 30˚C: hematoxylin for ∼10 min, eosin for 2 min). A 40× objective lens on a light microscope, integrated with a camera and computer interface, was employed to analyze the slides at a total magnification of 1000× [26]. The images of each specimen were analyzed by an expert unaware of the grouping. Histopathological alterations were semiquantitatively assessed using a scoring system evaluating the cellular injury.

### Molecular docking and ADMET properties

Proteins related to oxidative stress, inflammatory response, hepatic regeneration, and metabolic processes were chosen for docking purposes. These proteins include IL-6, TNF-α, peroxisome proliferator-activated receptor γ (PPAR-γ), superoxide dismutase (SOD), transforming growth factor-β 1 (TGF-β1), cytochrome P450 2C9 (CYP2C9), and cytochrome P450 2E1 (CYP2E1). IL-6 and TNF-α were chosen because they are key proinflammatory cytokines that are markedly upregulated after CCl_4_ exposure and contribute to inflammatory tissue damage and disease progression. PPAR-γ was selected for its roles in lipid metabolism, glucose homeostasis, and anti-inflammatory signaling, which are relevant to the observed restoration of lipid profile abnormalities. SOD was included as a representative antioxidant enzyme because oxidative stress and excessive reactive oxygen species generation are central mechanisms of CCl_4_-induced toxicity. TGF-β1 was selected because it is a major mediator of tissue fibrosis and extracellular matrix deposition during chronic liver and kidney injury [12,27]. CYP2E1 was investigated owing to its pivotal role in the bioactivation of CCl_4_ into highly reactive trichloromethyl radicals that initiate oxidative damage [28]. CYP2C9 was included because of its importance in hepatic xenobiotic metabolism and its potential involvement in the biotransformation of phytoconstituents [29]. The Protein Data Bank provides protein structures. PyMOL was used to remove heteroatoms, ligands, cofactors, metal ions, and water molecules from the protein structures to prevent interferences with ligand–protein interactions. Next, Swiss-PdbViewer 4.10 (https://spdbv.unil.ch/) was used to reduce protein energy. The processed proteins were loaded into the PyRx docking program [30]. The SDF files for the CA extract-derived chemicals were retrieved from the PubChem database. After importing the ligand molecules into the PyRx tool, docking was performed [31]. The grid box parameters for all proteins are listed in the Supplemental Table 1. The optimal position in terms of binding energy was reserved for visualization. The ligands and receptors were defined individually once the combination structures were imported into Biovia Discovery Studio (Dassault Systèmes). The absorption, distribution, metabolism, excretion, and toxicity (ADMET) properties of the best binding compounds were calculated from ADMET Lab 3.0 (https://admetlab3.scbdd.com/).

## Results

### GC-MS analysis

**FIGURE 1:**
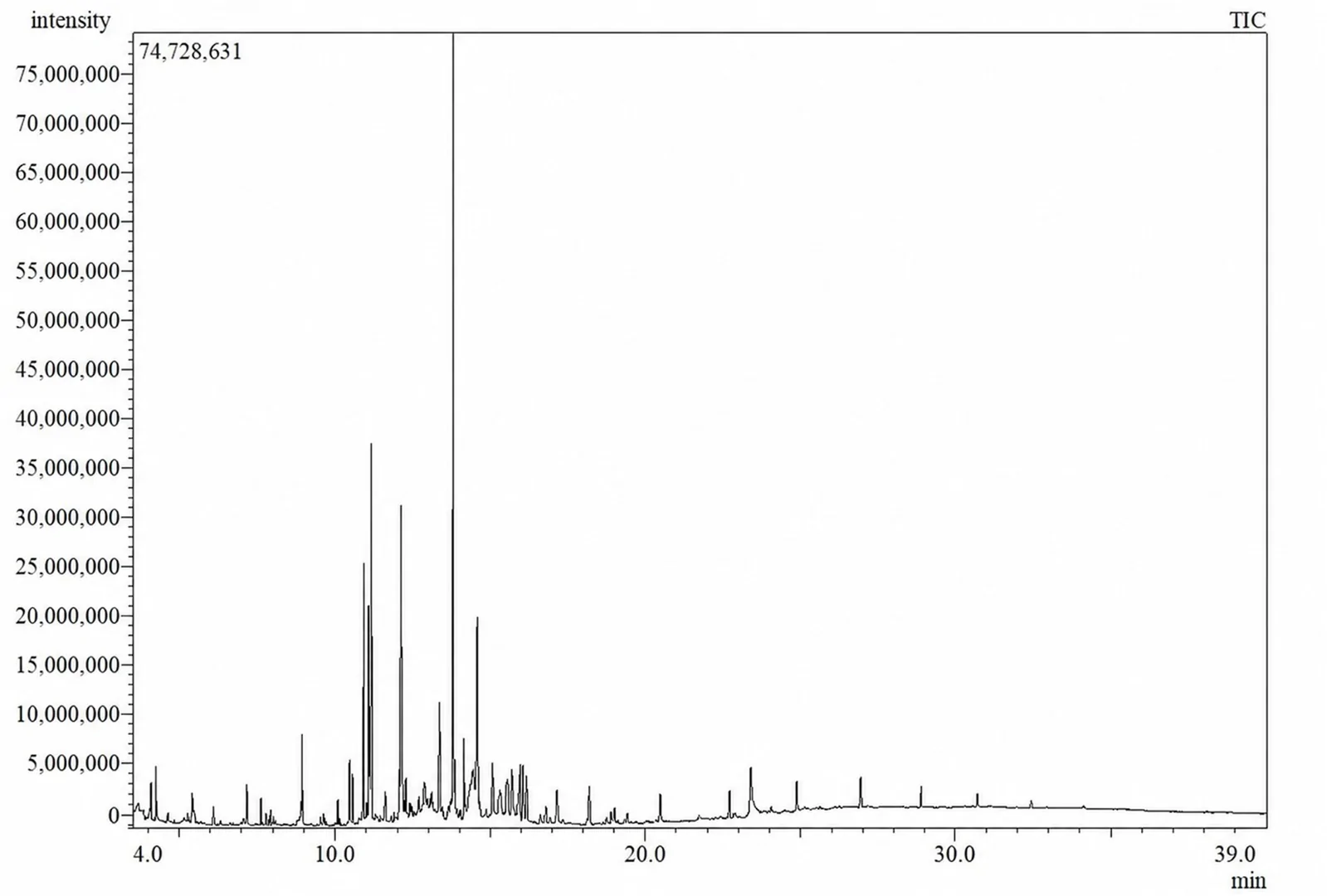
GC-MS chromatogram of *Curcuma amada* Roxb. extract.

The chromatogram of the *Curcuma amada* extract is given in Figure 1. The list of the most probable compounds is provided in Supplemental Table 2.

### Effect on liver biomarkers

**FIGURE 2:**
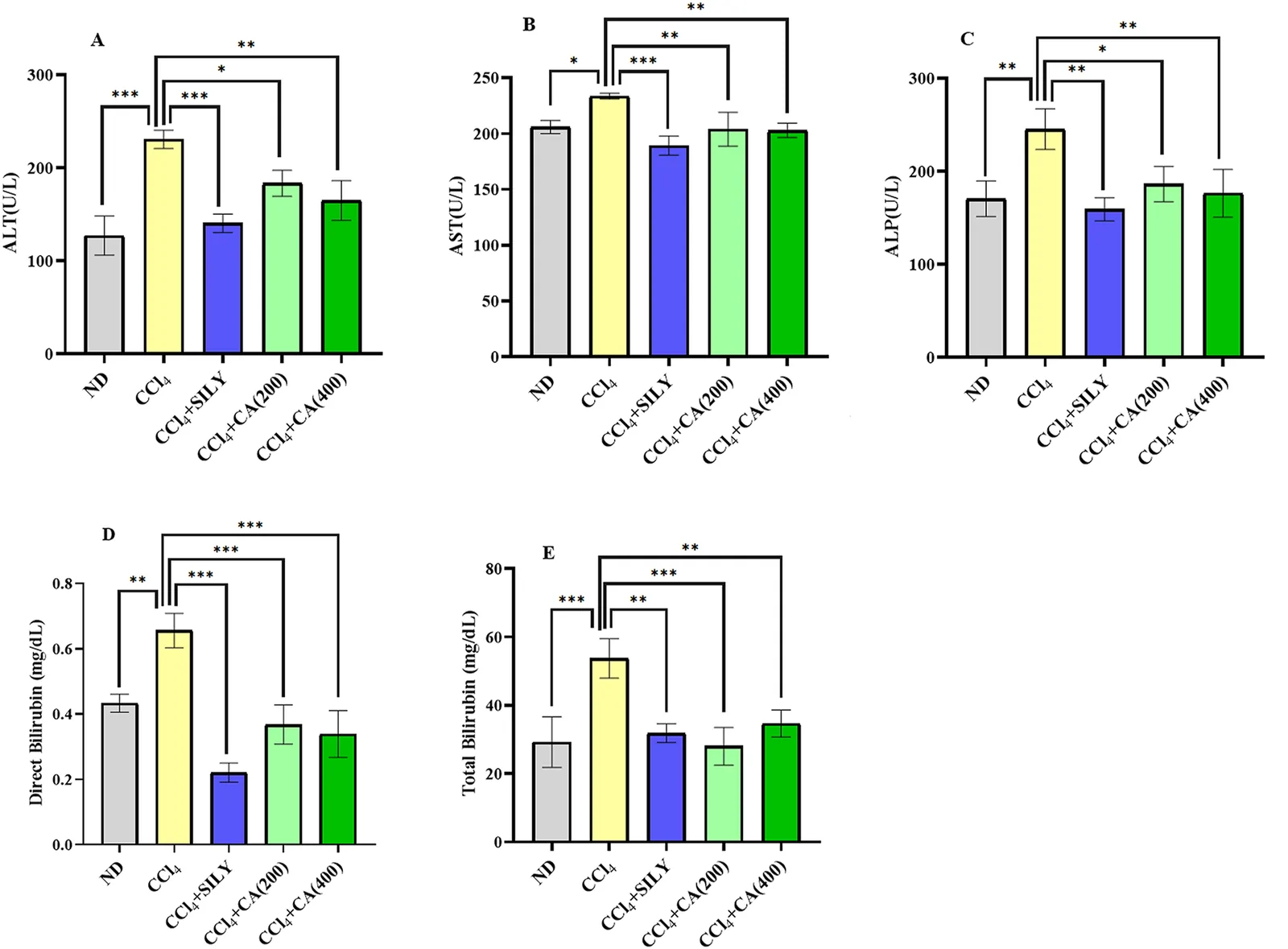
Effect of *Curcuma amada* Roxb. (CA) extract administration on liver enzymes of the mouse. (A) Alanine transaminase (ALT), (B) aspartate aminotransferase (AST), (C) alkaline phosphatase (ALP), (D) direct bilirubin, (E) total bilirubin. Data presented as mean ± SEM (*n* = 7), significantly different at ^∗^*P* < 0.05, ^∗∗^*P* < 0.01, ^∗∗∗^*P* < 0.001. CCl_4_, carbon tetrachloride; ND, normal diet; SILY, silymarin.

The effect of CA extract on liver biomarkers (ALT, AST, ALP, and bilirubin) is shown in Figure 2. Significant elevations in serum ALT, AST, ALP, direct bilirubin (DB), and total bilirubin (TB) levels were observed in the CCl_4_-treated group compared with the normal diet group (ALT: *P* ≤0.0001; AST: *P* = 0.0104; ALP: *P* = 0.0035; DB: *P* = 0.0012; TB: *P* = 0.0008). Compared with the CCl_4_-treated group, significant improvements were observed in the silymarin-treated groups (ALT: *P* = 0.0002; AST: *P* = 0.0004; ALP: *P* = 0.0013; DB: *P* ≤0.0001; TB: *P* = 0.0017), CA extract 200 mg/kg groups (ALT: *P* = 0.0157; AST: *P* = 0.0068; ALP: *P* = 0.0158; DB: *P* = 0.0002; TB: *P* = 0.0005), and CA extract 400 mg/kg groups (ALT: *P* = 0.0018; AST: *P* = 0.0054; ALP: *P* = 0.0060; DB: *P* ≤0.0001; TB: *P* = 0.0046).

### Effects on kidney markers

**FIGURE 3:**
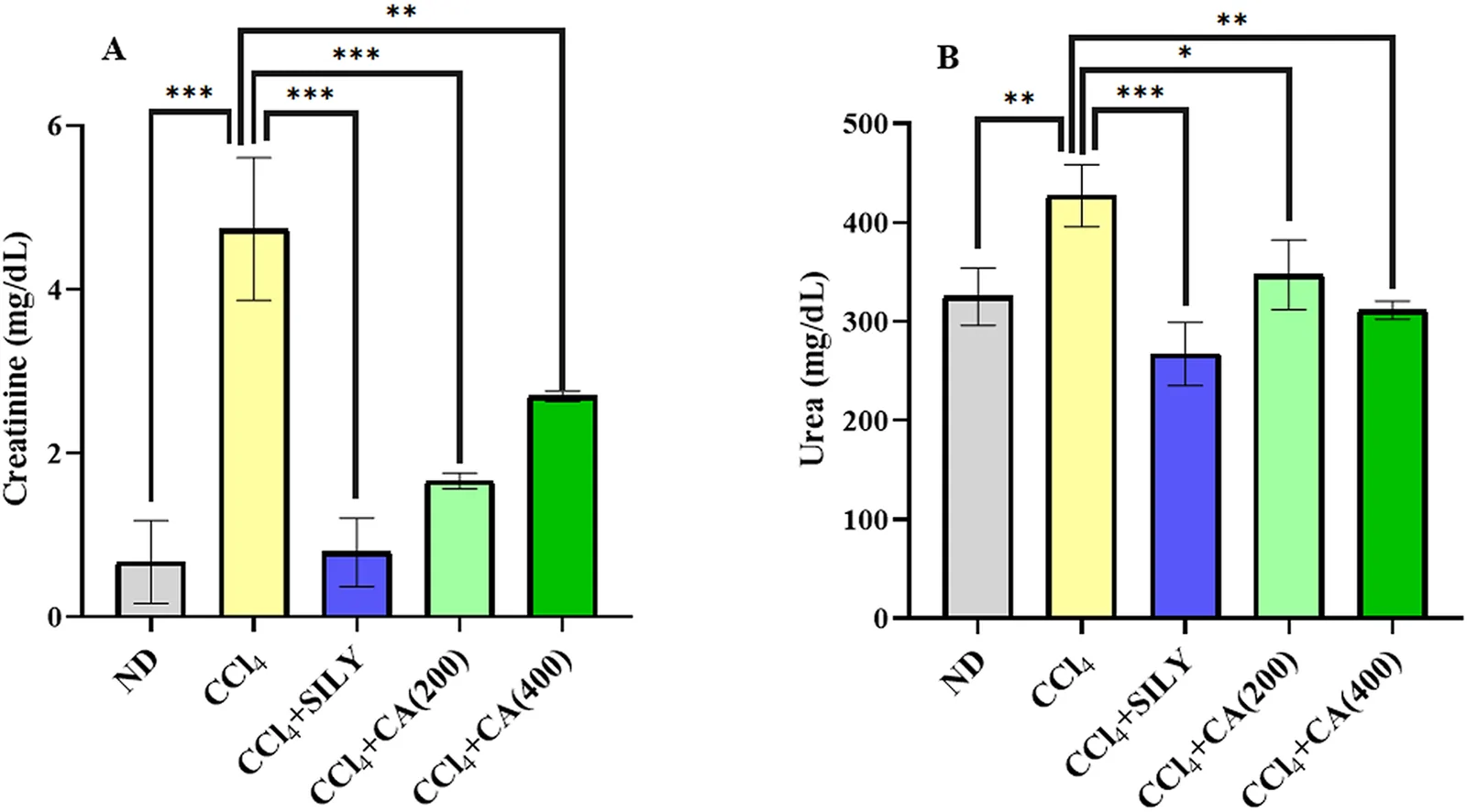
Effect of *Curcuma amada* Roxb. (CA) extract administration on kidney-associated markers of the mouse. (A) Creatinine, (B) urea. Data presented as mean ± SEM (*n* = 7), significantly different at ^∗^*P* < 0.05, ^∗∗^*P* < 0.01, ^∗∗∗^*P* < 0.001. CCl_4_, carbon tetrachloride; ND, normal diet; SILY, silymarin.

The administration of CCl_4_ in the experimental group of mice resulted in elevations in markers of kidney function (creatinine and urea). Figure 3 illustrates the effect of CA extract treatment on kidney biomarkers. Compared with the CCl_4_-treated group, significant improvements were observed in the silymarin-treated groups (creatinine: *P* ≤0.0001; urea: *P* = 0.0002), CA extract 200 mg/kg groups (creatinine: *P* ≤0.0001; urea: *P* = 0.0221), and CA extract 400 mg/kg groups (creatinine: *P* = 0.0016; urea: *P* = 0.0021).

### Effects on lipid levels

**FIGURE 4:**
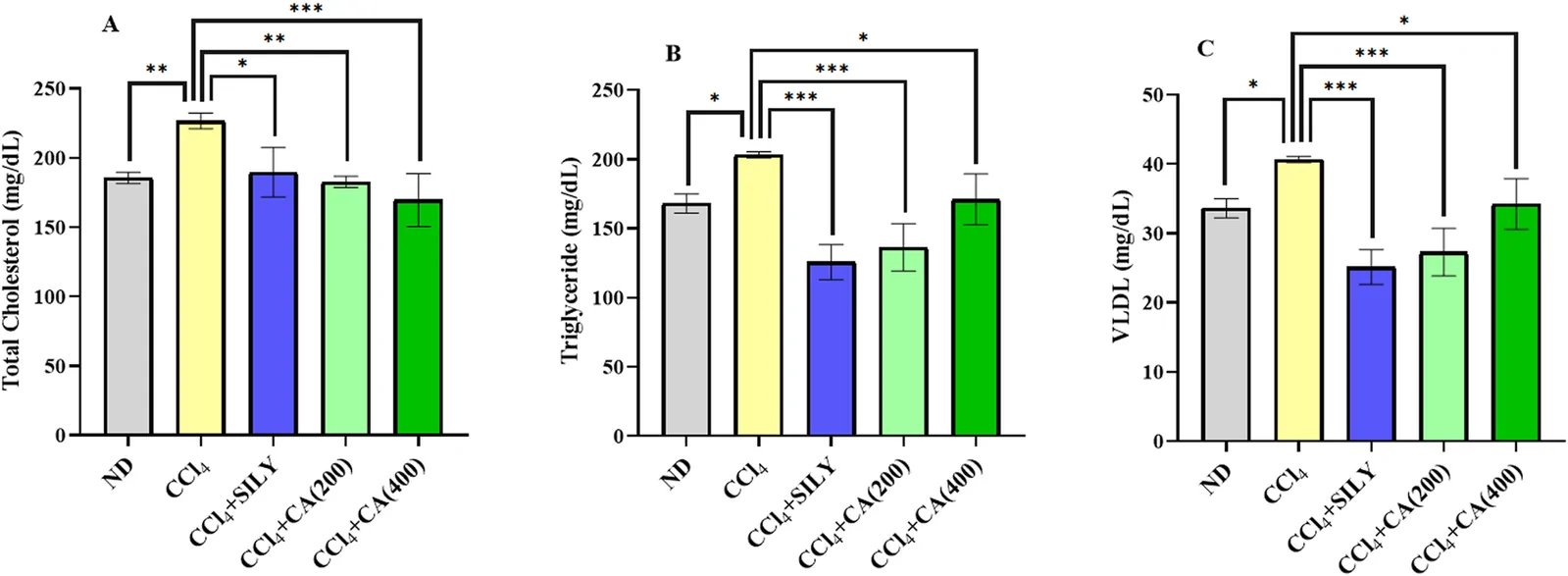
Effect of *Curcuma amada* Roxb. (CA) extract administration on lipid levels of the mouse. (A) Total cholesterol, (B) triglyceride, (C) VLDL. Data presented as mean ± SEM (*n* = 7), significantly different at ^∗^*P* < 0.05, ^∗∗^*P* < 0.01, ^∗∗∗^*P* < 0.001. CCl_4_, carbon tetrachloride; ND, normal diet; SILY, silymarin.

The CCl_4_ administration in the experimental group of mice caused an elevation of the serum lipid profile (Figure 4). Compared with the CCl_4_-treated group, significant improvements were observed in the silymarin-treated groups (TG: *P* = 0.0001; TC: *P* = 0.0132; VLDL: *P* = 0.0001), CA extract 200 mg/kg groups (TG: *P* = 0.0003; TC: *P* = 0.0044; VLDL: *P* = 0.0003), and CA extract 400 mg/kg groups (TG: *P* = 0.04; TC: *P* = 0.0007; VLDL: *P* = 0.04).

### Histopathological observation

**FIGURE 5:**
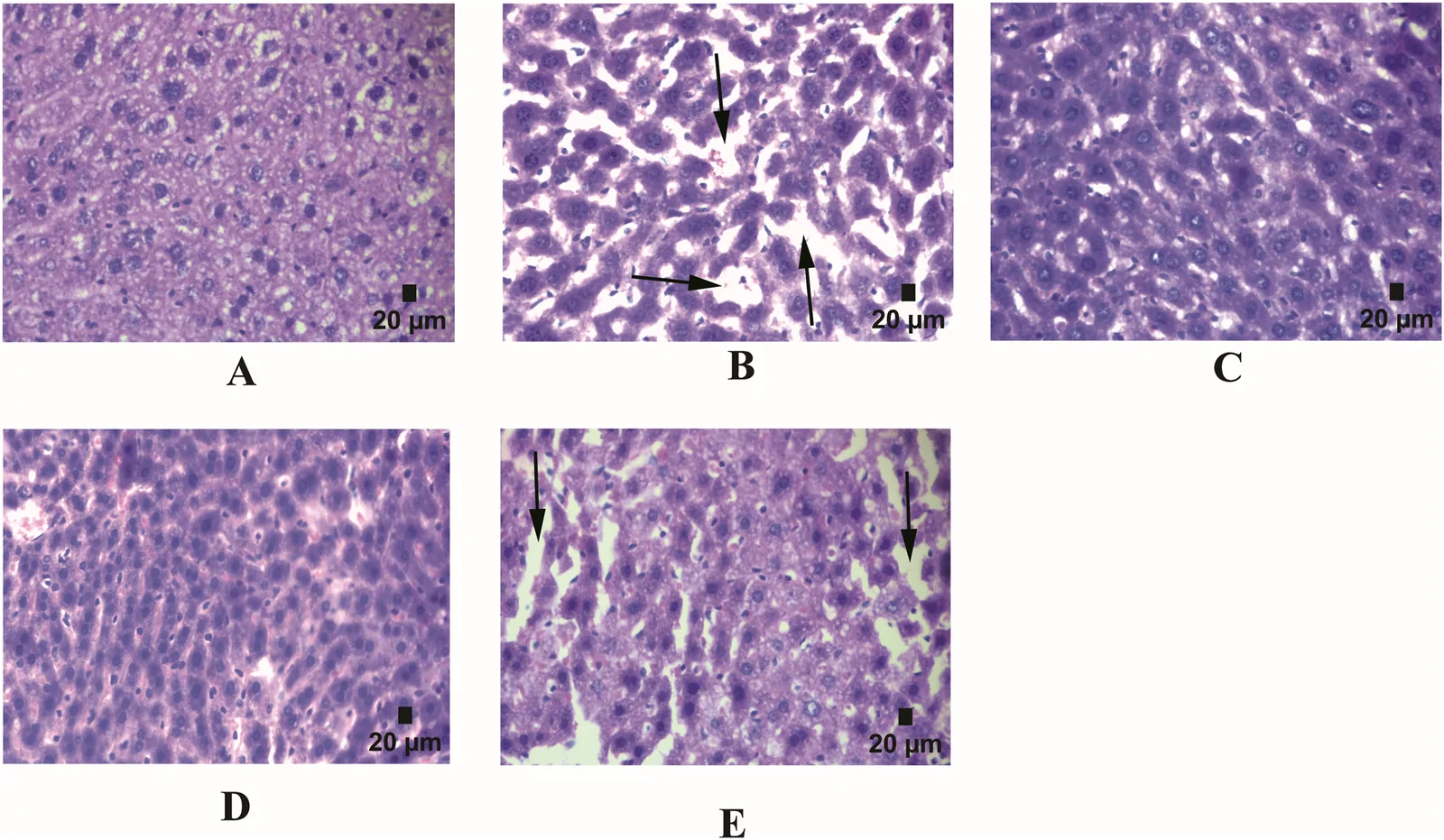
Effects of *Curcuma amada* Roxb. (CA) extract on the histopathological changes in the liver of mice treated with CCl_4_. (A) Normal diet, (B) disease control (CCl_4_-induced liver injury), (C) CCl_4_ + silymarin, (D) CCl_4_ + CA extract (200 mg/kg BW), (E) CCl_4_ + CA extract (400 mg/kg BW). BW, body weight; CCl_4_, carbon tetrachloride. Arrows denote cellular injury.

As shown in Figure 5, hepatocytes in the normal diet group were arranged in normal cords, with clear cytoplasm and well-defined nuclei without any necrotic foci. Oral administration of CCl_4_ led to visible damage in the liver due to severe hepatocellular degeneration, cytoplasmic vacuolization, necrosis, sinusoidal dilation, and inflammatory cell infiltration (Figure 5B). Noticeable regeneration of hepatocytes, reduced necrosis and inflammatory foci, and hepatic cords that appear nearly normal, with mild sinusoidal congestion, are visible in the silymarin group (Figure 5C). Moderate recovery, with partial restoration of hepatic architecture, fewer necrotic cells, a mild inflammatory state, and improved sinusoidal structure, is shown in the extract treatment (Figure 5D, E). Semiquantitative assessment was performed using a grading scale based on hepatocellular degeneration, steatosis, inflammatory cell infiltration, necrosis, and architectural disruption (Supplemental Table 3). The CCl_4_ group exhibited severe injury, whereas the silymarin- and extract-treated groups showed moderate injury.

**FIGURE 6:**
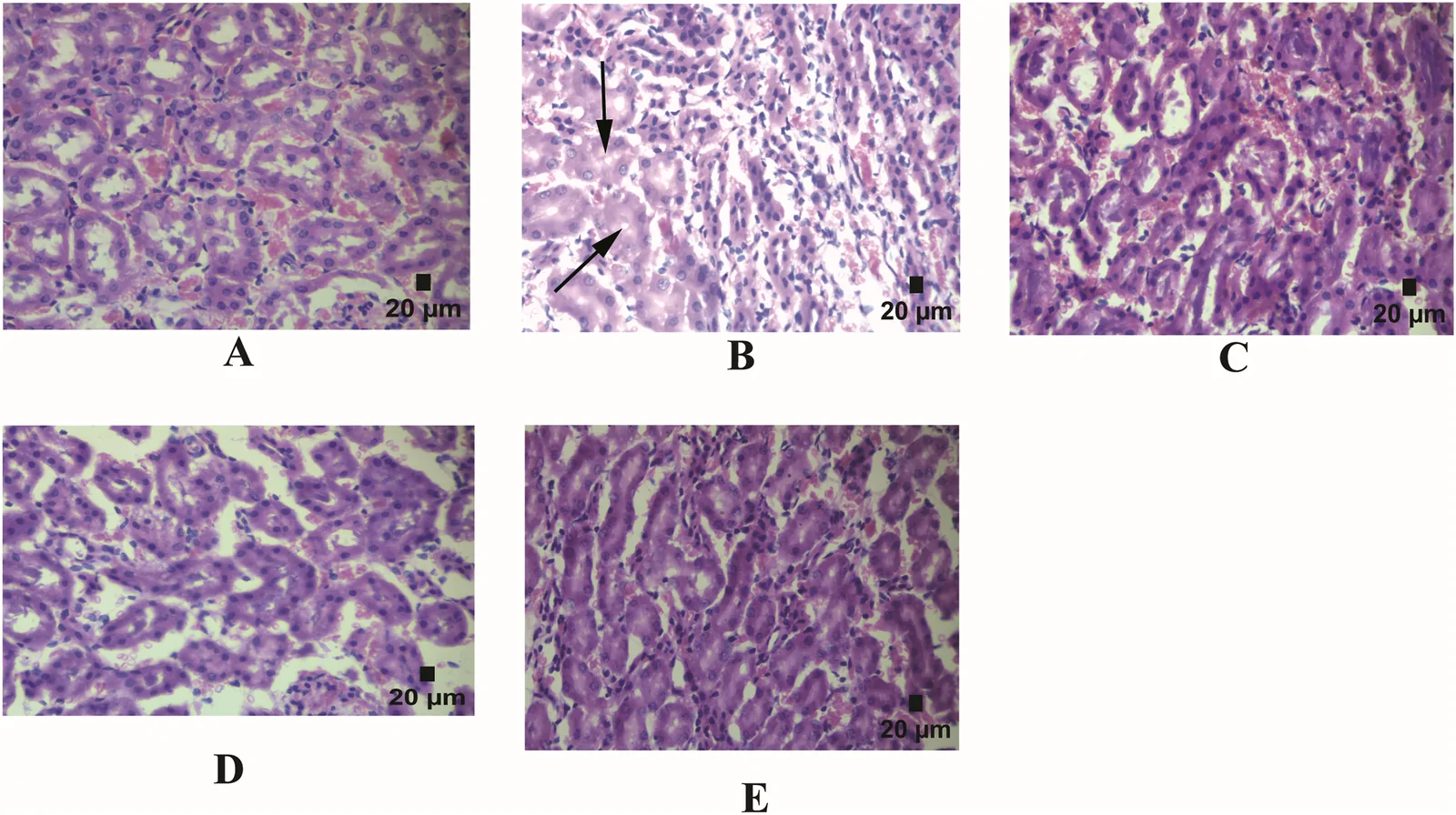
Effects of *Curcuma amada* Roxb. (CA) extract on the histopathological changes in the kidney of mice treated with CCl_4_. (A) Normal diet, (B) disease control (CCl_4_-induced injury), arrows denote cellular injury, (C) CCl_4_ + silymarin, (D) CCl_4_ + CA extract (200 mg/kg BW), (E) CCl_4_ + CA extract (400 mg/kg BW). BW, body weight; CCl_4_, carbon tetrachloride.

The histopathological findings of the kidney are shown in Figure 6. Figure 6A shows well-preserved renal corpuscles and tubular structures, an intact Bowman’s capsule, and uniform tubular epithelium. Pronounced glomerular atrophy, tubular necrosis, and cytoplasmic degeneration are shown in the CCl_4_ group (Figure 6B). Partial improvement, moderate tubular necrosis is observed in the silymarin group (Figure 6C). Significant restoration of renal tissue, with near-normal glomeruli and tubular integrity, minimal degenerative changes, and an inflammatory state, is shown in the extract-administered groups (Figure 6D, E). A semiquantitative assessment of the renal histopathological images showed severe renal injury for the CCl_4_ group. The silymarin and extract groups demonstrated mild-to-moderate renal injury (Supplemental Table 4).

### Molecular docking

The binding affinities of the compounds from the CA extract for proteins involved in oxidative stress, inflammation, and hepatic protection were calculated. The best binding affinities of the compounds with these proteins are listed in Table 1. Among the tested compounds, Psilostachyin B exhibited the most favorable binding energy with TNF-α, PPAR-γ, TGF-ß1, and CYP2C9. Similarly, curcumenol exhibited the most favorable binding energy with IL-6, 1H-Cycloprop[e]azulen-7-ol, decahydro-1,1,7-trimethyl-4-methylene- exhibited the most favorable binding energy with SOD, and methyl 10-*trans*,12-*cis*-octadecadienoate exhibited the most favorable binding energy with CYP2E1.

**TABLE 1:** Binding energies (kcal/mol) of selected compounds from CA with proteins related to inflammatory and hepatoprotective targets

Compounds (CID)	Proteins (PDB ID)						
	IL-6 (1ALU)	TNF-α (1TNF)	PPAR-γ (6MS7)	SOD (2C9V)	TGF-ß1 receptor (1VJY)	CYP2C9 (1OG2)	CYP2E1 (3LC4)
Psilostachyin B (5320768)	−6.8	−8.8	−8.9	−6.8	−8.5	−8.8	−7.9
Curcumenol (167812)	−7.0	−7.5	−8.1	−6.7	−7.7	−7.7	−8.0
1,3,6,10-Cyclotetradecatetraene, 3,7,11-trimethyl-14-(1-methylethyl)-, [S-(E,Z,E,E)]-(6436662)	−6.2	−6.6	−9.2	−6.3	−7.3	−8.3	−7.4
Methyl 10-*trans*,12-*cis*-octadecadienoate (5471014)	−4.5	−5.1	−5.7	−4.9	−6.5	−5.7	−8.4
1H-Cycloprop[e]azulen-7-ol, decahydro-1,1,7-trimethyl-4-methylene-, [1ar-(1a.α.,4a.α.,7.β.,7a.β.,7b.α.)]-(6432640)	−6.4	−7.3	−7.9	−7.6	−7.8	−7.4	−6.3
1,1,4,7-Tetramethyldecahydro-1H-cyclopropa(e)azulene-4,7-diol (178322)	−6.6	−7.5	−7.9	−6.9	−6.9	−7.9	−6.5
Longiverbenone (530428)	−6.3	−7.2	−7.8	−6.6	−6.3	−8.5	−6.7
6-Dehydropetasol (5275908)	−6.3	−6.3	−7.9	−6.5	−8.2	−7.3	−7.5
Linderane (6915739)	−6.5	−8.7	−8.6	−7.0	−7.8	−8.0	−6.9
3,5,8a-trimethyl-4,4a,8a,9-tetrahydronaphtho[2,3-b] furan (13874240)	−6.1	−7.4	−7.9	−6.7	−8.2	−7.9	−6.9

Abbreviations: CYP2C9, cytochrome P450 2C9; CYP2E1, cytochrome P450 2E1; PDB, Protein Data Bank; PPAR-γ, peroxisome proliferator-activated receptor γ; SOD, superoxide dismutase; TGF-β1, transforming growth factor-β 1.

**FIGURE 7:**
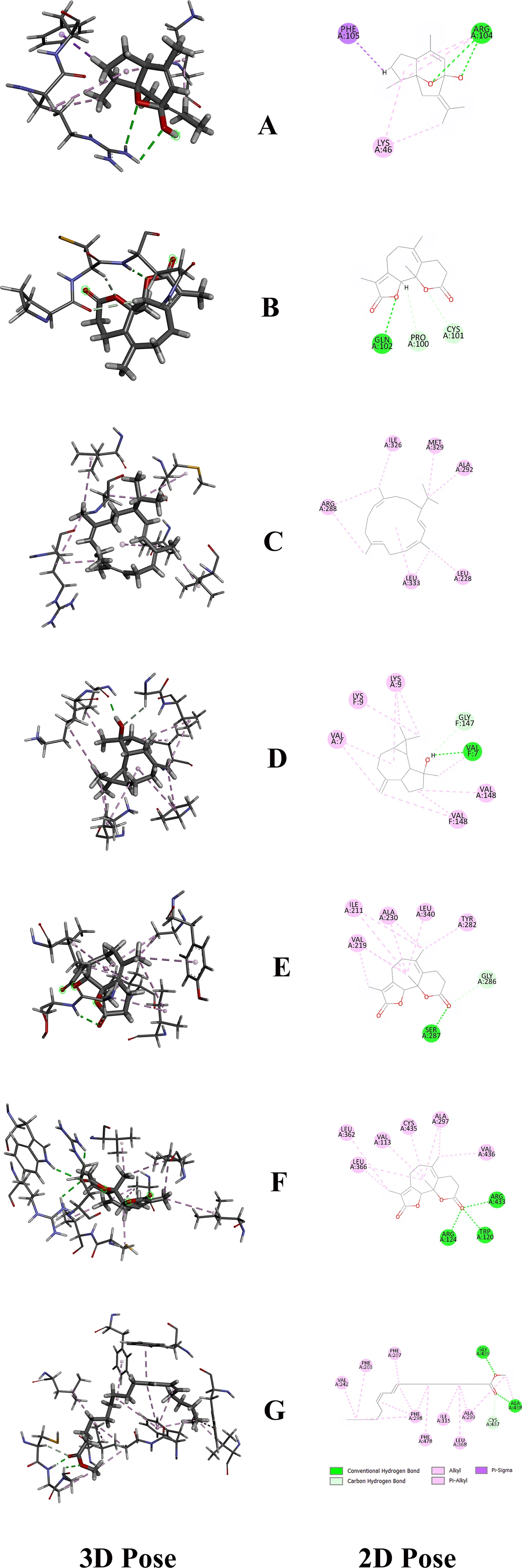
Possible 3-dimensional and 2-dimensional poses of selected compounds and proteins. (A) IL-6 and curcumenol, (B) TNF-α and psilostachyin B, (C) PPAR-γ and 1,3,6,10-cyclotetradecatetraene, 3,7,11-trimethyl-14-(1-methylethyl)-, [S-(E,Z,E,E)]-, (D) SOD and 1H-Cycloprop[e]azulen-7-ol, decahydro-1,1,7-trimethyl-4-methylene-, [1ar-(1a.α.,4a.α.,7.β.,7a.β.,7b.α.)]-, (E) TGF ß1 and psilostachyin B, (F) CYP2C9 and psilostachyin B, (G) CYP2E1 and methyl 10-*trans*,12-*cis*-octadecadienoate. CYP2C9, cytochrome P450 2C9; CYP2E1, cytochrome P450 2E1; PPAR-γ, peroxisome proliferator-activated receptor γ; SOD, superoxide dismutase.

The binding interactions of the best binding energies from each protein are shown in Figure 7. Figure 7A depicts the interaction of IL-6 with curcumenol. Curcumenol formed a conventional hydrogen bond with ARG104, a pi-sigma bond with PHE105, and an alkyl bond with LYS46. Psilostachyin B binds with TNF-α at GLN102 through a conventional hydrogen bond, CYS101, and PRO100 through a carbon–hydrogen bond (Figure 7B). Psilostachyin B forms alkyl bond at the ALA292, ARG288, ILE326, LEU288, LEU333, and MET329 positions of PPAR-γ (Figure 7C). Psilostachyin B displays strong interaction with TGF ß1 (Figure 7E). Psilostachyin B binds with a conventional hydrogen bond at SER287, a carbon–hydrogen bond at GLY287, an alkyl bond at ALA230, ILE211, LEU340, TYR282, and VAL219.

### ADMET profile

The ADMET properties of the top 10 scoring compounds are summarized in Table 2. The ranges signify the likelihood of the compound’s attributes. The ideal logP value spans from 0 to 3, whereas logS spans from −4 to 0.5. According to these criteria, the compounds with PubChem identifications 167812, 5275908, 5320768, and 6915739 are suitable candidates. The other compounds are outliers of the range and require modification. Hence, the solubility profile of these compounds must be modulated. Parameters linked to absorption generally indicated low probabilities of human intestinal absorption for most substances, with values ranging from 0.0001 to 0.026. The Caco-2 (human colon adenocarcinoma cell line) permeability values for most substances exceeded the optimum threshold of −5.15 log units. The probability of penetrating the blood–brain barrier (BBB) was too high for the compounds 530428, 5275908, 6432640, and 13874240. The plasma protein binding percentages indicate that chemicals 178322, 5275908, 5320768, and 6915739 exhibit binding values of <90%. The volume of distribution (0.285–4.778) and plasma clearance (5.569–14.198) values were optimal for all substances. Multiple compounds were identified as cytochrome P450 3A4 (CYP3A4) substrates, although only a few demonstrated CYP3A4 inhibitory capability. The likelihood of oral toxicity was minimal for most of the compounds. However, several compounds (5275908, 5320768, 6432640, 6915739, and 13874240) showed a higher degree of carcinogenicity in the ADMET analysis. These compounds must be assessed in subsequent stages of optimization and experimental validation. All the compounds complied with the Lipinski drug rules.

**TABLE 2:** ADMET properties of the selected compounds from *Curcuma amada* Roxb. extract

Properties	PubChem Compound ID number									
	167812	178322	530428	5275908	5320768	5471014	6432640	6436662	6915739	13874240
LogP	2.831	3.306	3.603	2.319	1.149	6.819	3.529	5.406	2.59	4.093
LogS	−2.291	−2.86	−3.825	−2.974	−2.795	−6.784	−3.271	−5.561	−3.443	−4.59
HIA (probability of HIA)	0.011	0.02	0.022	0.001	0.007	0.004	0.022	0.004	0.026	0.006
Caco-2	−4.609	−4.947	−4.868	−4.796	−4.961	−5.003	−5.062	−4.671	−4.492	−4.697
BBB (probability of being BBB+)	0.003	0.138	0.859	0.558	0.406	0.352	0.627	0.021	0.022	0.986
PPB (%)	93.20	59.10	93.50	67.50	89.20	97.80	93.60	97.50	84.70	94.80
V_D_ (L/kg)	2.408	1.155	1.038	0.914	1.491	0.285	0.852	2.062	0.871	2.591
CYP3A4 inhibitor	0.201	0.075	0.001	0.071	0.004	0.989	0.001	0.306	0.076	0.562
CYP3A4 substrate	0.715	0.84	0.998	0.02	0.208	0.145	0.071	0.006	0.954	0.991
C_L_ (mL/min/kg)	11.979	14.198	12.021	13.573	9.518	5.569	11.594	11.205	9.154	10.113
T_1/2_	1.878	2.4	0.666	1.974	1.416	0.408	1.739	0.43	1.53	0.557
Rat oral acute toxicity (probability of being toxic)	0.549	0.134	0.325	0.637	0.484	0.057	0.305	0.015	0.54	0.584
Carcinogenicity (probability of being carcinogenic)	0.555	0.693	0.686	0.841	0.937	0.127	0.857	0.046	0.797	0.741
Lipinski	Accepted	Accepted	Accepted	Accepted	Accepted	Accepted	Accepted	Accepted	Accepted	Accepted

Abbreviations: ADMET, absorption, distribution, metabolism, excretion, and toxicity; BBB, blood–brain barrier; Caco-2, human colon adenocarcinoma cell line; CYP3A4, cytochrome P450 3A4; HIA, human intestinal absorption; LogP, partition coefficient; LogS, solubility; PPB, plasma protein binding; T1/2, half-life.

## Discussion

The increasing global prevalence of liver and renal illnesses necessitates the immediate investigation of innovative treatment approaches. The quest for hepatoprotective and nephroprotective medicines from natural sources has intensified, propelled by the constraints and side effects of traditional pharmacotherapies [32]. Our study aimed to assess the therapeutic efficacy of an ethanolic extract of CA against CCl_4_-induced toxicity in a mouse model. In addition to hepatic injury, CCl_4_ exposure causes renal dysfunction through oxidative stress–mediated tubular necrosis, glomerular damage, and increased lipid peroxidation. It should also be noted that the present investigation employed a cotreatment design, in which CA extract was administered concurrently with repeated CCl_4_ exposure. Therefore, the findings demonstrate protective effects during ongoing toxicant exposure rather than prophylactic effects resulting from pretreatment before toxin administration. Our findings present strong evidence that treatment with CA offers substantial protection to both the liver and kidneys. The hepatotoxic and nephrotoxic effects of CCl_4_ are well-documented and widely used in experimental models. The reactive free radicals from CCl_4_ trigger a series of harmful events, including the peroxidation of membrane PUFAs, leading to compromised membrane integrity, organelle failure, and ultimately necrotic cell death [33]. This cellular attack is exacerbated by a vigorous inflammatory response, marked by immune cell infiltration and the secretion of proinflammatory cytokines, including TNF-α and IL-6, which further exacerbates tissue damage [34]. Our findings in the positive control group are entirely consistent with this recognized pathophysiology. The significant increase in serum levels of liver enzymes AST, ALT, and ALP is a traditional sign of hepatocellular injury and impaired plasma membrane integrity [35].

A critical finding of our research is the substantial, dose-dependent decrease in serum enzyme levels in the CA-treated groups. AST, ALT, and ALP levels were normalized to values akin to those of the silymarin-treated standard group. The increased bilirubin levels in the CCl_4_ control group indicate compromised absorption, conjugation, or excretion of bile pigments, typically due to significant hepatic dysfunction or bile duct damage [36]. The reduction in bilirubin levels after CA treatment indicates improved liver excretory capacity and overall functional recovery, further corroborating the extract’s holistic restorative action.

CCl_4_ intoxication significantly impairs hepatic lipid metabolism, resulting in the accumulation of triglycerides and cholesterol, which is characteristic of hepatic steatosis [37]. Our data unequivocally indicate that CCl_4_ treatment resulted in a substantial increase in serum TGs, TC, and estimated VLDL. Dyslipidemia results from compromised synthesis and release of apolipoproteins, diminished fatty acid β-oxidation, and increased lipogenesis [38]. The treatment of CA effectively countered this trend, aligning lipid profiles substantially with those of the normal control group. Our findings thus critically validate the antihyperlipidemic activity previously reported for CA in the context of toxin-induced metabolic dysfunction. Because excessive lipid accumulation, oxidative stress, and inflammation are major contributors to the pathogenesis of fatty liver disease, the observed lipid-equilibrating and hepatoprotective effects of CA extract may indicate its potential therapeutic relevance in fatty liver conditions. However, the current study was conducted using a CCl_4_-induced toxicity model rather than a dedicated fatty liver disease model. Consequently, the potential efficacy of CA extract against fatty liver disease remains speculative and requires further investigation.

The nephrotoxicity of CCl_4_, although often seen as after hepatotoxicity, may also arise from direct injury and systemic oxidative stress [39]. The kidneys are especially vulnerable to oxidative stress because of their high content of long-chain PUFAs, which are key targets for lipid peroxidation [40]. The marked elevation of serum urea and creatinine levels in the CCl_4_-treated group is a clear indicator of impaired renal function, reflecting a diminished glomerular filtration rate. Urea is the principal end-product of protein catabolism, whereas creatinine is a byproduct of muscle metabolism; healthy kidneys efficiently eliminate both. Their accumulation in the blood reflects functional impairment of the nephrons [41]. Cotreatment with CA resulted in a significant reduction in urea and creatinine levels. Although the biochemical findings are substantial, histological examination of liver and kidney tissues further supported the biochemical observation by demonstrating reduced necrosis, inflammatory infiltration, and tissue degeneration in the CA-treated groups. Both liver and kidney histology showed that CA extract protects against CCl_4_-induced injury. The finding aligns with previously reported hepatoprotective and nephroprotective effects of plant-derived polyphenolic extracts in a murine model [42,43].

Molecular docking of the chemical compounds from the CA extract exhibits strong binding affinities with the different proteins responsible for hepatoprotection, inflammation, and oxidative stress. Psilostachyin B showed the strongest binding energies with 3 proteins. The binding interactions revealed the types of chemical bonds with the amino acids of the selected proteins. These compounds showed favorable bonds with their receptor proteins. Molecular docking predicted favorable binding interactions between several phytochemicals and CYP-related proteins. However, docking analyses alone do not demonstrate modulation of CYP enzyme activity, and these predicted interactions require experimental validation. The in silico ADMET analysis revealed a mixed pharmacokinetic profile among the identified phytochemicals. Although several compounds complied with Lipinski’s rule of 5 and exhibited favorable predicted properties, others showed potential limitations, including poor predicted intestinal absorption, low aqueous solubility, high plasma protein binding, and relatively high probabilities of BBB penetration. In addition, some compounds demonstrated predicted toxicity-related signals that warrant careful consideration. Therefore, these compounds represent potential candidates for future investigation. However, their predicted pharmacokinetic limitations and possible toxicity-related signals indicate that additional experimental studies, including pharmacokinetic, toxicological, and efficacy evaluations, are required before their therapeutic potential can be established. An additional limitation of the present study is the absence of a vehicle-only control group. Although the experimental design enabled the evaluation of the protective effects of CA extract against CCl_4_-induced hepatorenal injury, it does not permit discrimination between the intrinsic physiological effects of the extract and its effects in the context of toxin-induced damage. Therefore, the observed biochemical and histopathological improvements should be interpreted as evidence of protection within the CCl_4_ injury model rather than as effects solely attributable to CA extract itself. Future studies incorporating a vehicle-only control group will be important to establish the independent biological effects and safety profile of the extract under normal physiological conditions. Furthermore, because no extract-only treatment group was included, the present study cannot evaluate the intrinsic safety, tolerability, or baseline biological effects of CA extract in healthy animals. Consequently, the observed absence of adverse outcomes in the treatment groups should not be interpreted as evidence of safety. Another limitation of this study is that, although animals were randomly allocated to the experimental groups, allocation concealment and complete investigator blinding were not implemented because the principal investigator remained aware of group assignments throughout the study. Only the histopathological assessment was performed under blinded conditions. Consequently, the possibility of performance or detection bias cannot be completely excluded.

Subsequent research should focus on bioassay-guided fractionation of CA to identify the specific molecule or synergistic combination of molecules responsible for the reported effects. A quantitative analysis of essential phytoconstituents, such as curcumin, demethoxycurcumin, and other phenolic acids, would facilitate the standardization of the extract. Investigating the specific molecular pathways, including the impacts on the Nrf2 (nuclear factor erythroid 2–related factor 2)–Keap1 (kelch-like ECH-associated protein 1) antioxidant system, nuclear factor κ B–mediated inflammation, and particular apoptotic markers such as caspase-3, will yield profound mechanistic insights [44].

In conclusion, the present study demonstrates that the ethanol extract of CA attenuated CCl_4_-induced hepatic and renal injury under the experimental conditions employed. Treatment with the extract improved altered liver enzymes, lipid levels, and renal functional markers compared with the disease group. Moreover, the extract ameliorated histopathological changes in liver and kidney tissues compared with the CCl_4_-treated group. The molecular docking and in silico ADMET analyses identified several phytochemicals with predicted interactions toward proteins associated with hepatorenal injury. However, the computational ADMET assessment also revealed potential pharmacokinetic and toxicity-related limitations for several compounds. Therefore, these findings should be considered hypothesis-generating and require comprehensive experimental validation before any conclusions regarding drug-likeness or therapeutic suitability can be drawn. Moreover, because the study did not include a vehicle-only control group, the independent physiological effects of *Curcuma amada* extract cannot be distinguished from its protective effects against CCl_4_-induced injury. Therefore, further investigations are required to elucidate the underlying molecular mechanisms, evaluate the intrinsic biological and toxicological effects of *Curcuma amada* extract using appropriate extract-only control groups, and validate its therapeutic potential in additional experimental models. Consequently, no conclusions regarding the inherent safety of the extract should be drawn from the present study.

## Author contributions

The authors’ responsibilities were as follows – M Mahmodul Islam, M Monirul Islam, MSH: designed research; SS, AAH: conducted research; M Mahmodul Islam: analyzed data; M Monirul Islam, SS: drafted the paper; MSH: primarily responsible for final content; and all authors: read and approved the final manuscript.

## Data availability

Data described in the manuscript, codebook, and analytic code will be made available upon request, pending.

## Funding

This work was partially funded by the Research Cell of Noakhali Science and Technology University.

## Declaration of generative AI and AI-assisted technologies in the writing process

During the preparation of this work, the author(s) used ChatGPT (free version) in order to improve the language and grammar. After using this tool/service, the author(s) reviewed and edited the content as needed and take(s) full responsibility for the content of the publication.

## Conflict of interest

The authors report conflicts of interest.
