# Supplementary material for: Protective Effects of Mango Ginger (Curcuma amada Roxb.) Ethanol Extract against Carbon Tetrachloride–Induced Toxicity: In Vivo and In Silico Evidence
Source: Curr Dev Nutr. 2026 Jul 31;10(9):109506. doi: 10.1016/j.cdnut.2026.109506 (PMC13522368; doi:10.1016/j.cdnut.2026.109506)
Supplement: Supplementary file 1 [file mmc1.docx]

Sagor Saha et al. Protective Effects of Mango Ginger (*Curcuma amada* Roxb.) Ethanol Extract Against Carbon Tetrachloride–Induced Toxicity: *In vivo* and *in silico* Evidence,

Supplementary file, S1

**Supplementary Table 1:** **The setup** **coordinates of grid boxes for molecular docking**

| Protein (PDB ID) | Coordinate (Å) | | | Size (Å) | | |
| --- | --- | --- | --- | --- | --- | --- |
|  | X | Y | Z | X | Y | Z |
| IL-6 (1ALU) | 2.5371 | -19.9863 | 9.0647 | 45.195479 | 49.757559 | 40.023599 |
| TNF-α (1TNF) | 20.0368 | 49.5566 | 40.0288 | 62.760391 | 63.931721 | 61.397229 |
| PPAR-γ (6MS7) | 46.3925 | 9.6024 | 18.4841 | 43.667350 | 47.870060 | 64.307290 |
| SOD (2C9V) | 20.0414 | -0.0371 | 13.9019 | 51.904908 | 70.193317 | 51.819158 |
| TGF ß1 (1VJY) | 11.9495 | 75.3145 | 12.2708 | 49.355670 | 63.574841 | 54.864718 |
| CYP2C9 (1OG2) | 36.0571 | 64.9823 | 25.315 | 102.384709 | 74.434437 | 75.316006 |
| CYP2E1 (3LC4) | -15.1941 | -20.1777 | 22.8079 | 98.303297 | 100.524356 | 70.922671 |

**Supplementary Table 2: The most probable compounds found from GC-MS analysis**

| S/N | R. Time | Area % | Compound name |
| --- | --- | --- | --- |
| 1 | 7.604 | 0.37 | (+)-2-Bornanone |
| 2 | 7.784 | 0.17 | Isoborneol |
| 3 | 8.078 | 0.14 | .alpha.-Terpineol |
| 4 | 9.533 | 0.1 | Cyclohexene, 4-ethenyl-4-methyl-3-(1-methylethenyl)-1-(1-methylethyl)-, (3R-trans)- |
| 5 | 10.08 | 0.3 | Cyclohexane, 1-ethenyl-1-methyl-2,4-bis(1-methylethenyl)-, [1S-(1.alpha.,2.beta.,4.beta.)]- |
| 6 | 10.458 | 1.05 | .gamma.-Elemene |
| 7 | 10.765 | 0.28 | Formic acid, decyl ester |
| 8 | 10.909 | 3.81 | Benzene, 1-(1,5-dimethyl-4-hexenyl)-4-methyl- |
| 9 | 10.98 | 0.15 | Caryophyllene oxide |
| 10 | 11.018 | 0.35 | Germacrene D |
| 11 | 11.084 | 3.82 | Benzofuran, 6-ethenyl-4,5,6,7-tetrahydro-3,6-dimethyl-5-isopropenyl-, trans- |
| 12 | 11.162 | 5.54 | (R)-1-Methyl-4-(6-methylhept-5-en-2-yl)cyclohexa-1,4-diene |
| 13 | 11.306 | 0.3 | Cedrene |
| 14 | 11.378 | 0.25 | 1-Acetyl-2-(2'-oxo-propyl)-cyclopentane |
| 15 | 11.465 | 0.19 | (1aR,4S,4aR,7R,7aS,7bS)-1,1,4,7-Tetramethyldecahydro-1H-cyclopropa[e]azulen-4-ol |
| 16 | 11.609 | 0.88 | 7-epi-cis-sesquisabinene hydrate |
| 17 | 11.809 | 0.11 | 1,5-Cyclodecadiene, 1,5-dimethyl-8-(1-methylethylidene)-, (E,E)- |
| 18 | 11.935 | 0.12 | Limonen-6-ol, pivalate |
| 19 | 11.99 | 0.1 | trans-Sesquisabinene hydrate |
| 20 | 12.13 | 7.78 | .beta.-Elemenone |
| 21 | 12.223 | 0.44 | Humulenol-II |
| 22 | 12.28 | 0.82 | Epicurzerenone |
| 23 | 12.337 | 0.19 | Benzene, (1-butylheptyl)- |
| 24 | 12.429 | 0.54 | (5.beta.,8.alpha.,9.beta.,10.alpha.,13.alpha.,16.beta.)-16-Hydroxy-17-((3-methylbutanoyl)oxy)kauran-18-oic acid |
| 25 | 12.481 | 0.41 | Isospathulenol |
| 26 | 12.553 | 0.31 | Guaiol |
| 27 | 12.81 | 0.53 | 1-Ethynylcyclododecanol |
| 28 | 12.887 | 1.44 | 7-epi-trans-sesquisabinene hydrate |
| 29 | 12.972 | 0.65 | 1H-Cycloprop[e]azulen-7-ol, decahydro-1,1,7-trimethyl-4-methylene-, [1ar-(1a.alpha.,4a.alpha.,7.beta.,7a.beta.,7b.alpha.)]- |
| 30 | 13.053 | 0.66 | Retinal |
| 31 | 13.128 | 0.85 | 1,3,6,10-Cyclotetradecatetraene, 3,7,11-trimethyl-14-(1-methylethyl)-, [S-(E,Z,E,E)]- |
| 32 | 13.205 | 0.26 | Benzene, (1-methyldecyl)- |
| 33 | 13.249 | 0.29 | Psilostachyin B |
| 34 | 13.359 | 2.55 | 3,7-Cyclodecadien-1-one, 3,7-dimethyl-10-(1-methylethylidene)-, (E,E)- |
| 35 | 13.455 | 0.63 | 3,5,8a-trimethyl-4,4a,8a,9-tetrahydronaphtho[2,3-b]furan |
| 36 | 13.645 | 0.42 | (4E)-11,11-Dimethyl-8-methylidenebicyclo[7.2.0]undec-4-ene-4-carboxylic acid, TMS |
| 37 | 13.719 | 0.78 | Curcumenol |
| 38 | 13.8 | 16.59 | Phenol, 2-methyl-5-(1,2,2-trimethylcyclopentyl)-, (S)- |
| 39 | 13.953 | 0.22 | (-)-Neoclovene-(I), dihydro- |
| 40 | 14.036 | 0.25 | Zederone |
| 41 | 14.161 | 2.02 | 6-Dehydropetasol |
| 42 | 14.225 | 0.36 | Silphiperfola-4,7(14)-diene |
| 43 | 14.361 | 1.5 | 1,1,4,7-Tetramethyldecahydro-1H-cyclopropa[e]azulene-4,7-diol |
| 44 | 14.445 | 2.25 | 5,8-Dihydroxy-4a-methyl-4,4a,4b,5,6,7,8,8a,9,10-decahydro-2(3H)-phenanthrenone |
| 45 | 14.593 | 5.4 | (3R,4aS,5R)-4a,5-Dimethyl-3-(prop-1-en-2-yl)-1,2,3,4,4a,5,6,7-octahydronaphthalene |
| 46 | 14.873 | 0.21 | Longiverbenone |
| 47 | 15.08 | 1.23 | Curcumenone |
| 48 | 15.28 | 0.34 | 1,2-Dehydro-.alpha.-cyperone |
| 49 | 15.333 | 0.79 | 4,5-Epoxygermacrone |
| 50 | 15.548 | 1.68 | Hydroxyvalerenic acid |
| 51 | 15.71 | 1.1 | Linderane |
| 52 | 16.066 | 1.13 | Naphthalene, 1,2,3,4-tetrahydro-1-methyl-8-(1-methylethyl)- |
| 53 | 16.177 | 1.22 | Hexadecanoic acid, methyl ester |
| 54 | 16.616 | 0.17 | .beta.-Cyclodihydrocostunolide |
| 55 | 16.806 | 0.39 | 2-Cyclohexen-1-one, 4-(3-hydroxy-1-butenyl)-3,5,5-trimethyl- |
| 56 | 17.165 | 0.84 | (1R,3aS,5aS,8aR)-1,3a,5a-Trimethyl-4-methylenedecahydrocyclopenta[c]pentalene |
| 57 | 18.118 | 0.15 | Trimethylsilyl 3-methoxy-2-(2-oxo-2-((trimethylsilyl)oxy)ethoxy)benzoate |
| 58 | 18.91 | 0.27 | Methyl 10-trans,12-cis-octadecadienoate |
| 59 | 19.01 | 0.38 | 9-Octadecenoic acid, methyl ester, (E)- |
| 60 | 19.419 | 0.35 | Methyl stearate |
| 61 | 23.398 | 1.82 | 9-Octadecenamide, (Z)- |
| 62 | 24.06 | 0.16 | Eicosyl isopropyl ether |

**Supplementary Table 3: Semi-quantitative scoring of liver histopathology**

| Group | Degeneration | Steatosis | Inflammation | Necrosis | Architecture | Overall Grade |
| --- | --- | --- | --- | --- | --- | --- |
| ND | 1 | 0 | 1 | 0 | 1 | Minimal |
| CCl_4_ | 2 | 1 | 3 | 2 | 3 | Severe |
| CCl_4_ + Silymarin | 2 | 2 | 2 | 1 | 2 | Moderate |
| CCl_4_ + CA 200 | 1–2 | 1 | 2 | 1 | 1 | Moderate |
| CCl_4_ + CA 400 | 2 | 1 | 2 | 1–2 | 2 | Moderate |

**Hepatocellular Degeneration:** 0 = Normal hepatocytes, 1 = Mild swelling / ballooning (<25%), 2 = Moderate (25–50%), 3 = Severe (>50%)

**Steatosis (Fat Accumulation):** 0 = None, 1 = <25% hepatocytes affected, 2 = 25–50%, 3 = >50%

**Inflammation (Portal + Lobular):** 0 = None, 1 = Mild scattered cells, 2 = Moderate infiltration, 3 = Dense inflammatory clusters

**Necrosis / Apoptosis:** 0 = None, 1 = Occasional cell death, 2 = Focal necrosis, 3 = Confluent necrosis

**Architectural Distortion:** 0 = Normal, 1 = Mild dilation, 2 = Moderate distortion, 3 = Severe disruption

**Supplementary Table 4: Semi-quantitative scoring of kidney histopathology**

| Group | Tubular Degeneration | Inflammatory cell infiltration | Necrosis | Overall renal injury | Interpretation |
| --- | --- | --- | --- | --- | --- |
| ND | 0 | 0 | 0 | 0 | Normal renal architecture |
| CCl_4_ | 4 | 4 | 3 | 4 | Severe renal injury with marked inflammation |
| CCl_4_ + Silymarin | 2 | 2 | 1 | 2 | Moderate renal injury |
| CCl_4_ + CA 200 | 1 | 1 | 1 | 2 | Moderate renal injury |
| CCl_4_ + CA 400 | 2 | 1 | 1 | 2 | Moderate renal injury |

**Scoring**: 0 = Normal histology, 1 = Mild lesion (<25% tissue involvement), 2 = Moderate lesion (25–50% tissue involvement), 3 = Marked lesion (50–75% tissue involvement), 4 = Severe lesion (>75% tissue involvement)
